# Microbiota-Influenced Toxicological Responses in *Caenorhabditis elegans* Exposed to Strawberry and Menthol E-Liquids

**DOI:** 10.17912/micropub.biology.001699

**Published:** 2025-08-11

**Authors:** Zahira Quinones Tavarez, Deborah J. Ossip, Dongmei Li, Daniel P. Croft, Irfan Rahman, Andrew P. Wojtovich

**Affiliations:** 1 Clinical Translational Science Institute, University of Rochester, Rochester, New York, United States; 2 Department of Public Health Sciences, University of Rochester, Rochester, New York, United States; 3 Department of Medicine, Pulmonary Diseases and Critical Care, University of Rochester Medical Center, Rochester, New York, United States; 4 Department of Environmental Medicine, University of Rochester Medical Center, Rochester, New York, United States; 5 Department of Anesthesiology and Perioperative Medicine, University of Rochester Medical Center, Rochester, New York, United States

## Abstract

Electronic nicotine delivery systems are marketed as safer than cigarettes, but their flavoring agents may be toxic. We evaluated reproductive effects of menthol- and strawberry-flavored e-liquids in *Caenorhabditis elegans* using wild-type and chemosensory-defective mutants ( *ocr-2 ; osm-9 ; ocr-1 ; trpa-1* ). L4-stage worms were exposed to flavored e-liquids on peptone-free media with *Escherichia coli* or natural microbiota ( *Lelliottia amnigena* , *Stenotrophomonas indicatrix* , *Comamonas piscis* ). Flavored exposure reduced brood size; menthol delayed egg-laying. Microbiota mitigated effects in most strains except *ocr-2 ; osm-9 ; ocr-1* with strawberry. Findings show flavored e-liquids harm reproduction, and microbiota may protect against flavor-induced toxicity.

**Figure 1. Effects of strawberry and menthol flavored e-liquids on brood size in wild type worms (WT) and chemosensory defective mutants (TRPV [ ocr-2, osm-9, ocr-1 ] and TRPA-1 [ trpa-1 ]) seeded on E. coli OP50 or members of the natural microbiota of C. elegans (CeMBio-3):**
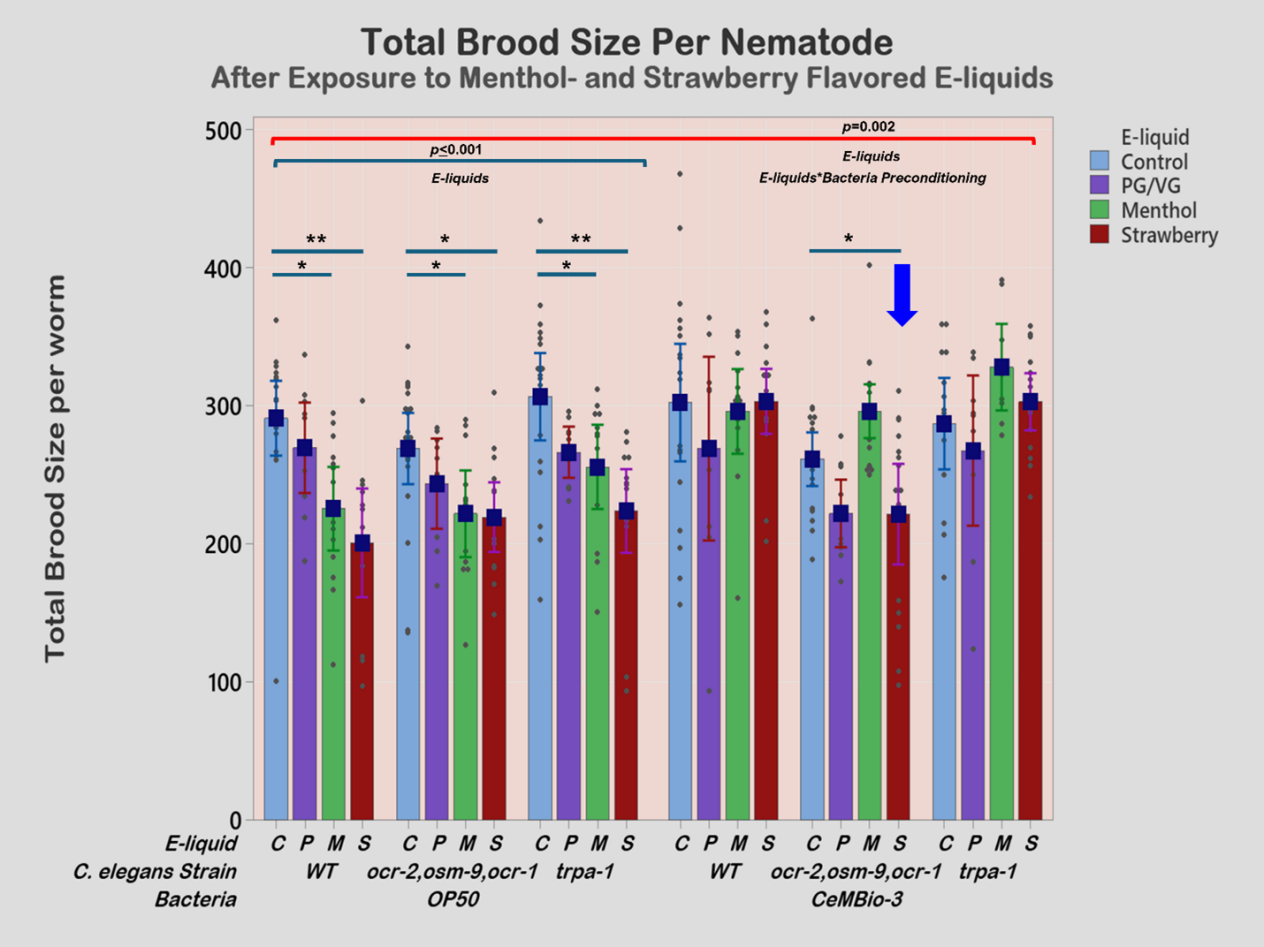
The red horizontal bracket shows the p-value from a two-way ANOVA test. The blue horizontal bracket shows results from one-way ANOVA. Dunnett tests were performed as post-test to compare groups against control. Significant differences between exposure groups and control are shown with p-values; non-significant group differences are omitted. Error bars represent 95% confidence intervals of the mean. Sample sizes (n) for each condition are as follows: for the three genetic variations of nematodes seeded on *E. coli OP50* , WT (control: n=18, PG/VG: n=10, menthol: n=14, strawberry: n=12), *ocr-2 , osm-9 , ocr-1* (control: n=20, PG/VG: n=9, menthol: n=12, strawberry: n=14), *trpa-1* (control: n=19, PG/VG: n=9, menthol: n=13, strawberry: n=15); and for nematodes seeded on members of the natural microbiota of *C. elegans* – WT (control: n=18, PG/VG: n=9, menthol: n=13, strawberry: n=16), *ocr-2 , osm-9 , ocr-1* (control: n=18, PG/VG: n=10, menthol: n=17, strawberry: n=16), *trpa-1* (control: n=14, PG/VG: n=9, menthol: n=9, strawberry: n=15).

## Description

Electronic nicotine delivery systems (ENDS) are often marketed as safer alternatives to cigarettes and as cessation aids, yet evidence for their long-term safety and efficacy remains limited. While ENDS may be less harmful than combustible tobacco, they are not risk-free. ENDS contain various toxic constituents—nicotine, solvents, humectants, and flavorings—which are linked to health effects similar to those caused by traditional cigarettes (Davis et al., 2022; Haswell et al., 2023; Kaur et al., 2018; Lee & Kim, 2023; Majek et al., 2023). Aerosols from ENDS contain lower but detectable concentrations of toxicants found in cigarette smoke, including carcinogens and reactive oxygen species (ROS) (Belkin et al., 2023; Czekala et al., 2019; Muthumalage et al., 2017; Tang et al., 2022) contributing to inflammation and oxidative stress.

Regulatory concerns have centered around flavors, which are linked to youth initiation, ongoing adult use, and misperceptions of harm (Cullen et al., 2019; Li et al., 2022; Romijnders et al., 2018). Studies show certain flavors—such as menthol, cinnamon, and strawberry—can enhance cellular toxicity by altering immune responses and disrupting epithelial barrier integrity (Gaurav, 2019; Johne et al., 2023), through the activation of transient receptor potential ankyrin 1 (TRPA1) and transient receptor potential vanillin 1 (TRPV1). Activation of these receptors by those flavorings contributes to inflammation and may be associated with adverse sensory or nociceptive effects (Bitar et al., 2025). However, few studies have specifically examined these outcomes as biological endpoints of ENDS flavor exposure (Muthumalage et al., 2019; Muthumalage et al., 2017). Although *in vitro* and animal models offer valuable insights, limited standardization across studies complicates interpretation and translational relevance (Muthumalage et al., 2024). Common findings include epithelial disruption, elevated pro-inflammatory signaling, and receptor-mediated cellular stress responses involving TRPA1 and TRPV1 (Lamb et al., 2020; Lamb & Rahman, 2023; Muthumalage et al., 2019). In this study, we examined the toxic effects of flavored e-liquids—specifically menthol and strawberry—on reproductive health in the nematode *Caenorhabditis elegans* ( *C. elegans* ), and the potential protective effects of its natural microbiota. *C. elegans* is a widely used model organism for toxicology, neurobiology, and host-microbe interaction studies due to its genetic tractability, transparent anatomy, and conserved molecular pathways (Markaki & Tavernarakis, 2020; Wu et al., 2019). Under laboratory conditions, *C. elegans* reproduces rapidly and allows high-throughput screening of developmental and reproductive toxicity (Xiong et al., 2017). Importantly, chemosensory pathways involving TRP channels, such as TRPA-1 and TRPV subunits ( OCR-2 , OSM-9 , OCR-1 ), play key roles in chemosensation and reproductive regulation (Panitz et al., 2015).

To assess toxicity, we exposed wild-type ( N2 Bristol) and TRP channel mutants ( *ocr-2 , osm-9 , ocr-1* triple mutant and *trpa-1* mutant) to menthol- and strawberry-flavored e-liquids on agar plates seeded with *E. coli* OP50 . Brood size and timing of egg-laying were used as outcome measures. Results showed that both flavors significantly reduced total brood size in wild-type worms ( *p<* 0.0001), with menthol exposure also delaying peak egg-laying to Day 2 (127.1±28.5 compared to 63.7±45.4 in the control group, *p <* 0.001). Vehicle controls using PG/VG showed no significant reproductive effects, isolating the toxicity to flavoring chemicals. Similar reproductive toxicity was observed in all mutant strains exposed to menthol and strawberry e-liquids. Total brood size was reduced by approximately 20–33% depending on genotype and flavor (p<0.05–0.001). Notably, menthol exposure caused delayed egg-laying in both mutant strains. Strawberry exposure led to sharp brood reductions in *trpa-1* mutants on Day 1, but not to the same delay seen with menthol.

We next evaluated whether the nematode's native microbiota could mitigate these toxic effects. Three bacterial strains from the CeMbio microbiota resource— *Lelliottia amnigena* , *Stenotrophomonas indicatrix* , and *Comamonas piscis* —were used to precondition and feed worms in place of *OP50* . Under this condition, wild-type and *trpa-1* mutants exposed to flavored e-liquids showed restored brood sizes, with no significant differences from controls. This suggests microbiota-mediated protection. However, *ocr-2 , osm-9 , ocr-1* mutants exposed to strawberry e-liquids still exhibited a significant reduction in brood size, indicating that TRPV channels may be necessary for the microbiota's protective effect. Daily reproductive counts supported these findings: wild-type and *trpa-1* mutants showed improved progeny production across days when fed microbiota strains, especially on Days 0–2. In contrast, *ocr-2 , osm-9 , ocr-1* mutants remained susceptible to strawberry e-liquid toxicity, even with microbiota preconditioning. These results suggest that TRP channels may modulate both flavor-induced toxicity and the protective effects of the microbiota. Previous studies support this idea—Panitz et al. showed decreased brood size and developmental delay in worms exposed to nicotine and PG (Panitz et al., 2015), while Wang et al. demonstrated lifespan reduction following flavored e-liquid exposure (Wang et al., 2020). Although neither study tested microbiota interactions, both support the harmful potential of e-liquid components.

Interestingly, our study used nicotine-free e-liquids, isolating flavor effects from nicotine-induced toxicity. The finding that PG/VG alone was not toxic further emphasizes the role of flavoring chemicals in reproductive impairment. This aligns with *in vitro* data showing that flavorings can independently induce oxidative and inflammatory responses (Muthumalage et al., 2019; Muthumalage et al., 2017). Given that PG/VG alone can elicit inflammatory responses, our findings suggest that the reproductive effects observed with flavored e-liquids may involve additional, possibly flavor-specific mechanisms—potentially independent of, or distinct from, the inflammatory pathways activated by PG/VG. Our findings also highlight the emerging importance of integrating microbiota into toxicological evaluations. Recent work by Haçariz et al. demonstrated that natural microbial strains colonizing the *C. elegans* gut enhanced resistance to toxicants such as juglone and silicon dioxide nanoparticles through activation of detoxification pathways (Haçariz et al., 2021). Similarly, in our study, microbiota preconditioning improved reproductive outcomes in most strains, potentially through modulation of metabolic or immune pathways.

In summary, this study demonstrates that menthol- and strawberry-flavored e-liquids impair reproduction in *C. elegans* via mechanisms likely involving TRPA1 and TRPV channels. Preconditioning with members of the natural microbiota can mitigate these effects in wild-type and *trpa-1* mutants, but not in *ocr-2 , osm-9 , ocr-1* mutants, suggesting TRPV-dependent protection. Our results provide foundational insights for using *C. elegans* in evaluating ENDS flavor toxicity and host-microbiota interactions. Future work integrating transcriptomic profiling and microbial colonization analysis will further elucidate mechanisms underlying microbiota-mediated resilience to environmental exposures.

## Methods

Nematode strains were obtained from the Caenorhabditis Genetics Center and were maintained at 20 °C on 60 mm NGM agar plates seeded with *E. coli* OP50 , with periodic transfers to prevent overcrowding and starvation. Three nematode strains were included in the study: N2 Bristol (wild type) and the chemosensory defective RB1052 , and FG125 . Age-synchronized, germ-free L4-stage nematodes were prepared by treating well-fed gravid hermaphrodites with an alkaline hypochlorite solution. Worms were washed off NGM plates using M9 buffer (3 g KH₂PO₄, 6 g Na₂HPO₄, 5 g NaCl, 1 mL 1 M MgSO₄, H₂O to 1 L) until the supernatant was clear. The worm pellet was then treated with bleach solution, followed by several washes with M9 buffer to remove residual bleach. Egg pellets were resuspended in 100 µL of sterile M9 buffer and plated onto 35 mm NGM peptone-free (NGMPF) dishes seeded with the bacteria of interest. NGMPF plates were utilized to minimize bacterial overgrowth during experiments.

Preparation of Bacterial Mixtures

Strains of bacteria utilized in this study were obtained from the Caenorhabditis Genetics Center and included the standard *E. coli OP50* and members of the CeMbio resource group (natural microbiota of *C. elegans* ) (Dirksen et al., 2020). The following bacterial strains were utilized in this study: *Lelliottia amnigena* ( JUb66 ), *Stenotrophomonas indicatrix* ( JUb19 ), *Comamonas piscis* ( BIGb0172 ). Upon arrival, single colonies from each bacteria culture plate were individually inoculated and cultured (48 hours) in Luria Broth medium at 25 °C and posteriorly stored at as 50% glycerol stocks at -80 °C to prevent bacteria adaptation to laboratory conditions. Natural microbiota mixtures were prepared by recovering bacterial cultures from glycerol stocks onto 60 mm LB agar plates (10 g Bacto-tryptone, 5 g Bacto-yeast, 5 g NaCl, 15 g agar, H₂O to 1 L, pH 7.5) and incubating for 48 h at 25 °C. Individual colonies were used to inoculate 800 µL of LB medium in 1 mL deep-well plates and incubated for 48 h at 25 °C. Bacterial growth was assessed via spectrophotometry and normalized to an OD₆₀₀ of 1.0 using sterile-filtered M9 buffer. Pellets from each bacterial culture were obtained by centrifugation, and a microbiota master mix was created by combining equal volumes of each strain in sterile tubes, according to experimental groups. All procedures were conducted under a biosafety cabinet.

Flavored E-liquids

Commercial flavored e-liquids were purchased in a local vaping shop in San Antonio, Texas. Strawberry flavors are among the most toxic and popular flavors preferred by users (Leigh et al., 2016) (Bitzer et al., 2018). The e-liquids contained no nicotine and a PG to VG ratio of 50:50. In the current study, a vehicle control group containing the same ratio of PG/VG was prepared using chemicals purchased from a vaping online store.

Exposure to Flavored E-liquids

NGMPF plates were prepared 24 h prior to exposure by adding strawberry or menthol flavored e-liquids at a concentration of 50 µL per mL of NGMPF media. This concentration was determined based on pilot acute toxicity assays, where a 50% lethality rate was observed in wild-type nematodes exposed to 50 µL of the original e-liquid solution. Control groups received either M9 buffer or a 50:50 PG/VG mixture. Exposure commenced upon the addition of eggs to the pretreated NGMPF plates seeded with the respective bacterial mixtures.

Brood Size Assay

For brood size assays, age-synchronized L4.4–L4.6 stage worms were individually transferred (n = 5 per treatment) onto new control or e-liquid pretreated NGMPF plates seeded with bacterial mixtures and maintained at 20 °C. Adult worms were transferred daily to new plates over a 5-day period, allowing egg-laying, and progeny were counted daily post-hatching. Each treatment condition included at least three biological replicates, with three technical iterations per experiment.

Statistical Analysis

Statistical analysis was performed with Minitab Statistical Software 21.1.0 (Minitab Inc., State College, PA, USA). Data for continuous variables are shown as mean ± standard error of means (SEM) if normally distributed; otherwise, median with 25 and 75 percentiles are provided. Kolmogorov-Smirnoff test was used to assess normality. One-way ANOVA and Dunnet post-test (normal distribution) or Kruskal-Wallis followed by Dunn's Test (non-normal data) were performed. Two-way ANOVA were performed to assess the effects of exposure to control/flavored e-liquid and type of bacterial preconditioning across *C. elegans* strains. Error bars represent the 95% confidence interval of the mean. p < 0.05 was considered for significant differences.

## Reagents

**Table:** 

Strain	Genotype	Source
RB1052	*trpa-1 ( ok999 )* IV (TRPA1 channel knockout)	Caenorhabditis Genetics Center (CGC)
FG125	*ocr-2 ( ak47 ) osm-9 ( ky10 )* IV; *ocr-1 ( ak46 )* V (TRPV channel triple knockout)	Caenorhabditis Genetics Center

## References

[R1] Belkin Svenja, Benthien Julia, Axt Paul Niklas, Mohr Theresa, Mortensen Kai, Weckmann Markus, Drömann Daniel, Franzen Klaas Frederik (2023). Impact of Heated Tobacco Products, E-Cigarettes, and Cigarettes on Inflammation and Endothelial Dysfunction. International Journal of Molecular Sciences.

[R2] Bitar Mariam, Mercier Clément, Bertoletti Laurent, Pourchez Jérémie, Forest Valérie (2025). Flavor-induced inflammation and cytotoxicity in human aortic smooth muscle cells: Potential implications for E-cigarette safety. Toxicology and Applied Pharmacology.

[R3] Bitzer Zachary T., Goel Reema, Reilly Samantha M., Elias Ryan J., Silakov Alexey, Foulds Jonathan, Muscat Joshua, Richie John P. (2018). Effect of flavoring chemicals on free radical formation in electronic cigarette aerosols. Free Radical Biology and Medicine.

[R4] Cullen Karen A., Gentzke Andrea S., Sawdey Michael D., Chang Joanne T., Anic Gabriella M., Wang Teresa W., Creamer MeLisa R., Jamal Ahmed, Ambrose Bridget K., King Brian A. (2019). e-Cigarette Use Among Youth in the United States, 2019. JAMA.

[R5] Czekala Lukasz, Simms Liam, Stevenson Matthew, Tschierske Nicole, Maione Anna G., Walele Tanvir (2019). Toxicological comparison of cigarette smoke and e-cigarette aerosol using a 3D in vitro human respiratory model. Regulatory Toxicology and Pharmacology.

[R6] Davis Lauren C., Sapey Elizabeth, Thickett David R., Scott Aaron (2022). Predicting the pulmonary effects of long-term e-cigarette use: are the clouds clearing?. European Respiratory Review.

[R7] Dirksen Philipp, Assié Adrien, Zimmermann Johannes, Zhang Fan, Tietje Adina-Malin, Marsh Sarah Arnaud, Félix Marie-Anne, Shapira Michael, Kaleta Christoph, Schulenburg Hinrich, Samuel Buck S (2020). CeMbio - The
*Caenorhabditis elegans*
Microbiome Resource. G3 Genes|Genomes|Genetics.

[R8] Gaurav Rohit (2019). Vaping Away Epithelial Integrity. American Journal of Respiratory Cell and Molecular Biology.

[R9] Haçariz Orçun, Viau Charles, Karimian Farial, Xia Jianguo (2021). The symbiotic relationship between Caenorhabditis elegans and members of its microbiome contributes to worm fitness and lifespan extension. BMC Genomics.

[R10] Haswell Linsey E., Gale Nathan, Brown Elaine, Azzopardi David, McEwan Michael, Thissen Jesse, Meichanetzidis Filimon, Hardie George (2023). Biomarkers of exposure and potential harm in exclusive users of electronic cigarettes and current, former, and never smokers. Internal and Emergency Medicine.

[R11] Johne Stephanie, van der Toorn Marco, Iskandar Anita R., Majeed Shoaib, Torres Laura O., Hoeng Julia, Peitsch Manuel C. (2023). An in vitro evaluation of e-vapor products: The contributions of chemical adulteration, concentration, and device power. Food and Chemical Toxicology.

[R12] Kaur Gurjot, Muthumalage Thivanka, Rahman Irfan (2018). Mechanisms of toxicity and biomarkers of flavoring and flavor enhancing chemicals in emerging tobacco and non-tobacco products. Toxicology Letters.

[R13] Lamb Thomas, Muthumalage Thivanka, Rahman Irfan (2020). Pod-based menthol and tobacco flavored e-cigarettes cause mitochondrial dysfunction in lung epithelial cells. Toxicology Letters.

[R14] Lamb Thomas, Rahman Irfan (2023). Pro-inflammatory effects of aerosols from e-cigarette-derived flavoring chemicals on murine macrophages. Toxicology Reports.

[R15] Lee Jae-woo, Kim Sukil (2023). Comparison of a Tobacco-Specific Carcinogen in Tobacco Cigarette, Electronic Cigarette, and Dual Users. Journal of Korean Medical Science.

[R16] Leigh Noel J, Lawton Ralph I, Hershberger Pamela A, Goniewicz Maciej L (2016). Flavourings significantly affect inhalation toxicity of aerosol generated from electronic nicotine delivery systems (ENDS). Tobacco Control.

[R17] Li Dongmei, Ossip Deborah J, Bansal-Travers Maansi, Xie Zidian (2022). Impact of the FDA flavour enforcement policy on flavoured electronic cigarette use behaviour changes. Tobacco Control.

[R18] Majek Paulina, Jankowski Mateusz, Brożek Grzegorz Marek (2023). Acute health effects of heated tobacco products: comparative analysis with traditional cigarettes and electronic cigarettes in young adults. ERJ Open Research.

[R19] Markaki Maria, Tavernarakis Nektarios (2020). Caenorhabditis elegans as a model system for human diseases. Current Opinion in Biotechnology.

[R20] Muthumalage Thivanka, Lamb Thomas, Friedman Michelle R., Rahman Irfan (2019). E-cigarette flavored pods induce inflammation, epithelial barrier dysfunction, and DNA damage in lung epithelial cells and monocytes. Scientific Reports.

[R21] Muthumalage Thivanka, Noel Alexandra, Thanavala Yasmin, Alcheva Aleksandra, Rahman Irfan (2024). Challenges in current inhalable tobacco toxicity assessment
models: A narrative review. Tobacco Induced Diseases.

[R22] Muthumalage Thivanka, Prinz Melanie, Ansah Kwadwo O., Gerloff Janice, Sundar Isaac K., Rahman Irfan (2018). Inflammatory and Oxidative Responses Induced by Exposure to Commonly Used e-Cigarette Flavoring Chemicals and Flavored e-Liquids without Nicotine. Frontiers in Physiology.

[R23] Panitz Daniel, Swamy Harsha, Nehrke Keith (2015). A C. elegans model of electronic cigarette use: Physiological effects of e-liquids in nematodes. BMC Pharmacology and Toxicology.

[R24] Romijnders Kim A. G. J., Van Osch Liesbeth, De Vries Hein, Talhout Reinskje (2018). Perceptions and Reasons Regarding E-Cigarette Use among Users and Non-Users: A Narrative Literature Review. International Journal of Environmental Research and Public Health.

[R25] Tang Moon-shong, Lee Hyun-Wook, Weng Mao-wen, Wang Hsiang-Tsui, Hu Yu, Chen Lung-Chi, Park Sung-Hyun, Chan Huei-wei, Xu Jiheng, Wu Xue-Ru, Wang He, Yang Rui, Galdane Karen, Jackson Kathryn, Chu Annie, Halzack Elizabeth (2022). DNA damage, DNA repair and carcinogenicity: Tobacco smoke versus electronic cigarette aerosol. Mutation Research/Reviews in Mutation Research.

[R26] Wang Ying, Ingram Thomas L, Marshall Sophie, Shephard Freya, Chakrabarti Lisa (2020). The effect of E-liquid exposure on
*Caenorhabditis elegans*.

[R27] Wu Tianshu, Xu Hongsheng, Liang Xue, Tang Meng (2019). Caenorhabditis elegans as a complete model organism for biosafety assessments of nanoparticles. Chemosphere.

[R28] Xiong Huajiang, Pears Catherine, Woollard Alison (2017). An enhanced C. elegans based platform for toxicity assessment. Scientific Reports.

